# Biliary Bacteria as an Indicator of the Risk of Recurrence of Choledocholithiasis After Endoscopic Sphincterotomy

**DOI:** 10.1155/DTE.5.9

**Published:** 1998

**Authors:** Jun Ishiguro

**Affiliations:** 1 Third Department of Internal Medicine Toho University School of Medicine Ohashi Hospital 2-17-6 Ohashi, Meguro-ku Tokyo 153 Japan

## Abstract

Bacteria have been implicated in recurrent choledocholithiasis associated with endoscopic sphincterotomy (EST). This study was designed to clarify whether bacterial examination of bile provides information useful in predicting the risk of recurrence of choledocholithiasis in patients undergoing EST. Bacteria in bile collected via a duodenoscope before cholangiography were cultured. We compared bacterial isolates and quantity among 41 patients with choledocholithiasis (7 with and 34 without a history of recurrent choledocholithiasis) who had undergone EST more than 3 months previously and 13 control patients with no evidence of pancreatobiliary disease. The bile samples were cultured under aerobic and anaerobic conditions. The bacterial quantity was expressed as the mean logarithm of the number of colony forming units (CFU)/ml. Furthermore, cholescintigraphic studies of bile flow were performed with the use of ^99 m^TC-HIDA to study the clinical implication of these variables. No bacteria were detected in 10 of the 13 patients in the control group. In the other three control patients the bacterial count was 2.2 log CFU/ml or less. The mean bacterial count was significantly higher in patients with recurrence than in those without recurrence. Cholescintigraphy revealed a trend toward a higher number of isolates and a higher bacterial count in bile in patients with delayed bile passage than in those with good passage. The results suggest that an increased number of biliary isolates and an increased bacterial count indicate decreased bile flow in patients with choledocholithiasis who are being followed up after EST. These variables may potentially serve as indicators of the risk of stone recurrence. Especially when the bacterial count is higher than 7.0 log CFU/ml, the risk of a decrease in bile flow and an increased stone recurrence would be possibly found.


*Diagnostic and Therapeutic Endoscopy*, Vol. 5, pp. 9–17
Reprints available directly from the publisher
Photocopying permitted by license only

© 1998 OPA (Overseas Publishers Association) N.V.
Published by license under
the Harwood Academic Publishers imprint,
part of The Gordon and Breach Publishing Group.
Printed in Singapore.

### Biliary Bacteria as an Indicator of the Risk of Recurrence of Choledocholithiasis after Endoscopic Sphincterotomy

JUN ISHIGURO *

*Third Department of Internal Medicine, Toho University School of Medicine, Ohashi Hospital, 2-17-6 Ohashi, Meguro-ku, Tokyo 153, Japan*

*(Received 20 October 1997; In final form 5 March 1998)*

**Bacteria have been implicated in recurrent choledocholithiasis associated with endoscopic sphincterotomy (EST). This study was designed to clarify whether bacterial examination of bile provides information useful in predicting the risk of recurrence of choledocholithiasis in patients undergoing EST. Bacteria in bile collected via a duodenoscope before cholangiography were cultured. We compared bacterial isolates and quantity among 41 patients with choledocholithiasis (7 with and 34 without a history of recurrent choledocholithiasis) who had undergone EST more than 3 months previously and 13 control patients with no evidence of pancreatobiliary disease. The bile samples were cultured under aerobic and anaerobic conditions. The bacterial quantity was expressed as the mean logarithm of the number of colony forming units (CFU)/ml. Furthermore, cholescintigraphic studies of bile flow were performed with the use of $^{99m}$TC-HIDA to study the clinical implications of these variables. No bacteria were detected in 10 of the 13 patients in the control group. In the other three control patients the bacterial count was 2.2 log CFU/ml or less. The mean bacterial count was significantly higher in patients with recurrence than in those without recurrence. Cholescintigraphy revealed a trend toward a higher number of isolates and a higher bacterial count in bile in patients with delayed bile passage than in those with good passage. The results suggest that an increased number of biliary isolates and an increased bacterial count indicate decreased bile flow in patients with choledocholithiasis who are being followed up after EST. These variables may potentially serve as indicators of the risk of stone recurrence. Especially when the bacterial count is higher than 7.0 log CFU/ml, the risk of a decrease in bile flow and an increased stone recurrence would be possibly found.**

*Keywords:* Bacteria in the bile duct, Recurrent choledocholithiasis, Cholescintigraphy

### 1. INTRODUCTION

Endoscopic sphincterotomy (EST) was introduced to general clinical practice about 20 years ago [1–3]. It is now routinely used in the treatment of choledocholithiasis. Many aspects of the long-term outcome of EST remain unknown, although several studies have addressed this problem [4–7].

* Tel.: 03-3468-1251. Fax: 03-3468-1269.

One late complication of EST is the recurrence of cholecystitis and choledocholithiasis. The incidence of cholecystitis has been estimated to range from 8.6% to more than 25% [4–7], and various measures have been proposed for the management of this complication.

Recurrence of choledocholithiasis is generally caused by calcium bilirubinate stones, the formation of which involves the bacterial flora, parapapillary diverticulum, and dietary habits [8]. $\beta$-glucuronidases produced by bacteria play a particularly important part in the formation of calcium bilirubinate stones [9–13]. Clinically, most cases of choledocholithiasis occur in elderly patients. Some asymptomatic cases are discovered incidentally on routine examination, but the majority of cases are symptomatic, often associated with severe symptoms of cholangitis. The identification of cases at high risk for recurrence requires regular follow-up by ultrasonography or endoscopic retrograde cholangiopancreatography (ERCP).

We studied bile bacteria before and after EST to test the hypothesis that bacterial flora in bile plays an important part in recurrence of choledocholithiasis. The primary objective was to determine whether biliary bacteria can serve as a prognostic factor indicating the risk of recurrent choledocholithiasis. In addition, cholescintigraphy was performed to study the clinical implications of related variables.

### 2. SUBJECTS AND METHODS

### 2.1. Subjects

The study group comprised 41 patients with choledocholithiasis who underwent EST. Thirty-four of these patients who underwent EST during the 22 months between August 1994 and June 1996 had no recurrence for at least 3 months after stone removal, and 7 patients had recurrence of choledocholithiasis. As control, 13 patients with no abnormalities of the bile ducts or pancreatic duct on ERCP were also studied. Thus, a total of 54 patients participated in the study (Table I). Although the duration of follow-up was short in the 34 patients with no recurrence and there was a risk of subsequent recurrence, in this study these patients were defined as having no recurrence. The 7 patients with recurrence were enrolled from among 206 patients with choledocholithiasis in whom lithotomy was performed by EST at Toho University Ohashi Hospital between October 1985 and March 1996. All patients had had a recurrence of choledocholithiasis and had undergone endoscopic lithotomy a second time. They were being followed up, and the mean duration of follow-up was 54.6 months. The mean period until recurrence was 32.2 months.

TABLE I Subjects

| | Cases | Age (mean) |
|---|---|---|
| Choledocholithiasis | 41 | 73.9 ± 9.8 |
| Recurrence | 7 | 75.3 ± 8.5 |
| Non-recurrence | 34 | 74.8 ± 9.8 |
| (before EST + after EL + Follow-up period) | (17) | |
| Control | 13 | 69.0 ± 5.3 |
| Total | 54 | 72.1 ± 9.4 |

Recurrence: with history of recurrence (August 1994–June 1996). Non-recurrence: without history of recurrence.

EST was performed via a medium or large incision. Transnasal biliary drainage was performed until the completion of lithotomy to prevent cholestasis [14]. During the procedure, all patients were given the same antibiotic, in principle. The common-bile-duct stones were analyzed by infrared spectroscopy to identify their principal components. The stones were classified according to the criteria proposed by the proposal of the Gallstone Study Group of the Japanese Society of Gastroenterology [15]. Among the patients with no recurrence, 19 had calcium bilirubinate stones and 15 had cholesterol stones. All seven patients with recurrence had calcium bilirubinate stones. We excluded patients with mixed stones consisting of at least 70% cholesterol and 5% or less of bilirubinate as well as those with black stones, which are considered to be caused by other factors. The mean age of the 41 patients with choledocholithiasis was $73.9 \pm 9.8$ years. Those with no recurrence were aged $74.8 \pm 9.8$, and those with recurrence $75.3 \pm 8.5$ years. The mean age of the control group was $69.0 \pm 5.3$ years. There was no significant difference in age among these groups.

### 2.2. Methods

Before enrollment the objectives of the study were explained to the patients. Informed consent was obtained from all patients before ERCP and taking samples. The endoscopes used for EST (JF200, JF230, JF1T30, Olympus optical co., Tokyo, Japan) were washed mechanically and allowed to dry naturally in a storage cabinet. A newly washed scope was used in each patient. Before ERCP a catheter sterilized with ethylene oxide gas was selectively placed in the common bile duct, and a minimum of 4 ml of bile was sampled. The bile samples were plated on CELD medium plates, Columbia Agar with 5% Sheep Blood plates, Chocolate Agar plates, and Anaero Columbia Agar with Rabbit Blood plates, which were then incubated under aerobic and anaerobic conditions. The resulting bacteria were identified on the basis of colony shape, microscopic examination of gram-staining properties, and the results of various biochemical tests. The lower detection limit was less than $2.0 \times 10^1$ CFU/ml (0.3 log CFU/ml). The number of CFU was measured quantitatively, and the total quantity of bacteria was expressed as the logarithm of the number of CFU/ml. If multiple species of bacteria were detected in the bile, any bacteria found in concentrations $\geq 1.0 \times 10^2$ CFU/ml lower than that of the predominant organism were considered negligible and were excluded. The bacteria count and quantity were thus determined for each specimen.

Bile samples were taken after endoscopic lithotomy when all clinical signs and symptoms of cholangitis had resolved and serum chemical tests showed normal values for biliary enzymes. Therefore, bile samples were taken at least 3 months after EST (follow-up). In the control group, bile samples were obtained only once before imaging studies during ERCP. In 17 of the patients without recurrence, bile samples were taken a total of three times (once before EST, once immediately after EST, and once before ERCP imaging studies) to study the time course of bile bacteria after EST.

Cholescintigraphy with $^{99m}$TC-dimethylephenylecarbamoyl-methyl iminodiacetic acid ($^{99m}$TC-HIDA) was performed at follow-up in a total of 21 patients, consisting of 19 without recurrence and 2 with recurrence, to assess biliary excretion. Patients with liver dysfunction due to liver disease or other conditions potentially affecting bile flow were excluded from the study.

Student's *t*-test and the Kruskal–Wallis test were used for statistical analysis. Differences with $P$ values of less than 0.05 were considered significant.

### 3. RESULTS

### 3.1. Bile Bacteria in the Control Group

Bacterial concentrations were below the detection limit of $2.0 \times 10^1$ CFU/ml (0.3 log CFU/ml) in 10 of the 13 subjects in the control group and were therefore not detected (Table II). Among the three other control subjects, a total of four species were identified: one species each in two patients and two species in one patient. The quantities of all bacteria were less than 2.2 log CFU/ml.

TABLE II Control cases

| Case (no.) | Bacterial | |
|---|---|---|
| | Isolates | Quantity (log CFU/ml) |
| 1–10 | | <0.3 |
| 11 | α-haemolytic Streptococcus | 2.2 |
| | Corynebacterium species | 1.3 |
| 12 | Enterocobacter cloacae | 1.3 |
| 13 | Staphylococcus epidermidis | 1.3 |

### 3.2. Bacteria in the 17 Patients Undergoing Examinations at Different Times

Before EST Enterococcus was the most frequent isolate, followed by E. coli and Klebsiella. Enteric bacteria (Enterobacilli), including primarily gram-negative bacilli were the most common isolates. After lithotomy and the resolution of clinical signs and symptoms of inflammation in response to antibiotic treatment, there was a trend toward a decrease in these gram-negative bacilli, but Enterococcus was detected in similar levels, and Streptococcus and Pseudomonas showed slight increases. On follow-up examination, E. coli and Kiebsiella, which had decreased transiently after lithotomy, reappeared. Anaerobes were first detected on follow-up examination (Table III).

Before EST the mean number of isolates detected was 2.4 ± 1.3, and the bacteria count was 5.4 ± 2.2 log CFU/ml. In patients with clinical evidence of cholangitis, the mean number of isolates was 2.9 ± 1.2 and the mean bacterial count was 6.2 ± 2.0 log CFU/ml. These values were higher than those in patients without cholangitis. After lithotomy and the resolution of clinical signs and symptoms of cholangitis, bile bacterial culture revealed the presence of 2.6 ± 1.8 isolates on average and a mean bacterial count of 5.1 ± 2.2 log CFU/ml. On average, bile samples were first taken 13 days after lithotomy. The mean period from lithotomy to the taking of follow-up bile samples was 103 days (Table IV). At that time the mean number of isolates was 3.1 ± 1.7, and the mean bacterial count was 4.8 ± 1.8 log CFU/ml.

TABLE III Bacterial isolates ($n = 17$)

| | Before EST | After EL | Follow-up |
|---|---|---|---|
| E. coli | 5 | 3 | 4 |
| Klebsiella | 5 | 2 | 6 |
| Enterobacter | 1 | 3 | 4 |
| Serratia | 4 | 2 | 1 |
| Aeromonas | | | 1 |
| Enterococcus | 10 | 11 | 4 |
| Streptococcus | 4 | 8 | 6 |
| Xantomonas | | | |
| Pseudomonas | 4 | 7 | 8 |
| Corynebacterium | 2 | 1 | 1 |
| Staphylococcus | | 3 | |
| Neisseria | | 1 | |
| Candida | 2 | | 2 |
| Clostridium | | 1 | |
| Peptostreptococcus | | | |
| Fusobacterium | | | |
| Bacteroides | | | 1 |
| Others | 1 | 2 | 5 |

EST: Endoscopic sphinterotomy. EL: Endoscopic lithotomy.

TABLE IV Mean number of bacterial isolates and quantity in each period ($n = 17$)

| | Isolates | Quantity (log CFU/ml) |
|---|---|---|
| Before EST | 2.4 ± 1.3 | 5.4 ± 2.2 |
| Cholangitis (+) ($n = 9$) | 2.9 ± 1.2 | 6.2 ± 2.0 |
| Cholangitis (–) ($n = 8$) | 1.9 ± 1.2 | 4.5 ± 2.1 |
| After EL | 2.6 ± 1.8 | 5.1 ± 2.2 |
| Follow-up period | 3.1 ± 1.7 | 4.8 ± 1.8 |

EL: endoscopic lithotomy (August 1994–June 1996).

### 3.3. Types and Frequencies of Bacterial Isolates in Patients with Recurrence and those without Recurrence (at Follow-up)

In both groups Enterobacilli including Enterococcus and gram-negative bacilli such as Klebsiella and E. coli were the most frequent isolates (Table V). In patients without recurrence, Streptococcus, Enterobacter, and Pseudomonas were frequently detected. In patients with recurrence, Klebsiella was detected at a rate of 85.7%, and E. coli was detected at a rate of 42.9%. Multiple isolates were found in 100% of patients with recurrence and 80.6% of patients without recurrence. E. coli was concurrently present in 40% and Klebsiella in 80% of the patients with recurrence. One species of anaerobe was detected in the patients with recurrence and three species were found in the patients without recurrence, but there was no significant difference between the groups.

The mean number of bacterial isolates was 2.8 ± 1.6 in the patients without recurrence and 3.7 ± 1.3 in the patients with recurrence (Table VI).

The mean bacterial count was significantly higher in the patients with recurrence (7.1 ± 0.7 log CFU/ml) than those without recurrence (4.9 ± 1.8 log CFU/ml) ($p < 0.05$; Table VII).

TABLE V Bacterial isolates

| | Nonrecurrence ($n=34$) | Recurrence ($n=7$) |
|---|---|---|
| E. coli | 11 (32.4) | 3 (42.9) |
| Klebsiella | 11 (32.4) | 6 (85.7) |
| Enterobacter | 5 (14.7) | |
| Serratia | 5 (14.7) | |
| Aeromonas | 3 (8.8) | |
| Enterococcus | 9 (26.5) | 3 (42.9) |
| Streptococcus | 13 (38.2) | 1 (14.3) |
| Xantomonas | 4 (11.7) | |
| Pseudomonas | 7 (20.6) | 3 (42.9) |
| Corynebacterium | 4 (11.7) | |
| Staphylococcus | 1 (2.9) | |
| Citrobacter | 2 (5.9) | 1 (14.3) |
| Morganella | 1 (2.9) | 1 (14.3) |
| Candida | 3 (8.8) | |
| Clostridium | 1 (2.9) | |
| Fusobacterium | | 1 (14.3) |
| Bacteroides | 2 (5.9) | |
| Others | 4 | 4 |

*Note:* Values in parentheses are in percentage.

TABLE VI Mean number of bacterial isolates more than 3 months after EST

| | Bacterial isolates |
|---|---|
| Non-recurrence ($n=34$) | $2.8 \pm 1.6$ |
| Bilirubin ($n=19$) | $3.2 \pm 1.9$ |
| Cholesterol ($n=15$) | $2.2 \pm 1.0$ |
| Recurrence ($n=7$) | $3.7 \pm 1.3$ |

Bilirubin: calcium bilirubinate stone.
Cholesterol: cholesterol stone.

TABLE VII Mean bacterial quantity more than 3 months after EST

| | Bacterial quantity (log CFU/ml) |
|---|---|
| Non-recurrence ($n=34$) | $4.9 \pm 1.8$ ┐ |
| Bilirubin ($n=19$) | $5.3 \pm 2.1$ │ |
| Cholesterol ($n=15$) | $4.3 \pm 1.5$ │* ┐ |
| Recurrence ($n=7$) | $7.1 \pm 0.7$ ┘ ┘ * |

Bilirubin: calcium bilirubinate stone.
Cholesterol: cholesterol stone.
$^{*}p < 0.05$, by Student's $t$-test.

### 3.4. Association of Bile Flow with Bacterial Isolates (at Follow-up)

The association of bile flow with bacterial isolates in bile was studied in a total of 21 patients (19 without recurrence and 2 with recurrence) who underwent $^{99m}$TC-HIDA cholescintigraphy. Passage of bile from the common bile duct to the duodenum within 20 min was defined as good passage, that requiring from 20 min to less than 45 min as slightly delayed passage, and that requiring 45 min or more as delayed passage [16]. Both of the patients with recurrence had delayed passage.

The mean number of isolates was $4.0 \pm 2.2$ in the patients with delayed passage and $2.6 \pm 1.1$ in the patients with good passage. The mean bacterial quantity was $7.4 \pm 1.3$ log CFU/ml and $3.2 \pm 1.1$ log CFU/ml, respectively. The bacterial count in patients with delayed passage was significantly higher than those in the other two groups ($p < 0.01$, 0.05; Table VIII). As for individual isolates (Table IX), among 7 patients with delayed passage, E. coli was isolated from 5 patients (71.4%), and Klebsiella was isolated from 6 patients (85.7%), as compared with rates of 0%

TABLE VIII Cholescintigraphic evaluation of bile flow and the mean number of bacterial isolates and quantity

| Cholescintigraphic findings | Bacterial | |
|---|---|---|
| | Isolates | Quantity (log CFU/ml) |
| Good ($n=8$) | $2.6 \pm 1.1$ | $3.2 \pm 1.1$ ┐ |
| Slightly delayed ($n=6$) | $2.7 \pm 0.9$ | $5.8 \pm 0.7$ ┐* │** |
| Delayed ($n=7$) | $4.0 \pm 2.2$ | $7.4 \pm 1.3$ ┘ ┘ |

Good: good passage. Slightly delayed: slightly delayed passage.
Delayed: delayed passage.
$^{*}p < 0.05$, by Student's $t$-test.
$^{**}p < 0.01$, by Student's $t$-test.

TABLE IX Cholescintigraphic evaluation of bile flow and bacterial isolates

| Cholescintigraphic findings | E. coli | Klebsiella | Anaerobic | Complex |
|---|---|---|---|---|
| Good ($n=8$) | 0 (0) | 2 (25) | 0 (0) | 6 (75) |
| Slightly delayed ($n=6$) | 1 (16.7) | 3 (50) | 0 (0) | 5 (83.3) |
| Delayed ($n=7$) | 5 (71.4) | 6 (85.7) | 3 (42.9) | 6 (100) |

Anaerobic: Anaerobic bacteria.
Good: good passage. Slightly delayed: slightly delayed passage.
Delayed: delayed passage.
*Note:* Values in parentheses are in percentage.

and 25% in patients with good passage and 16.7% and 50.0% in those with slightly delayed passage.

### 4. DISCUSSION

Recurrence of choledocholithiasis frequently involves calcium bilirubinate stones [17–19]. These stones are apparently formed through the action of β-glucuronidases produced by bacteria, which convert conjugated bilirubin, a water soluble substance, into insoluble free bilirubin, which binds with calcium and is deposited [9–13]. In addition to β-glucuronidases produced by E. coli, those elaborated by aerobes and anaerobes are also important. In mixed infections involving E. coli, a trend toward potentiation of β-glucuronidase activity has been reported [9–13].

Clinically, the recurrence rate of common-bile-duct stones has been studied by various investigators. Estimated recurrence rates range from 4.6% to up to 16% [3,5,17]. Takegawa *et al.* [20] reported a high recurrence rate of 16% and a mean interval of 35 months until recurrence. Although some of the differences are related to the duration of follow-up, in our study the recurrence rate was about 8.0% for a follow-up period ranging from 6 to 72 months (mean, 30.8 months). Generally, most cases of common-bile-duct stones recur between 2.5 and 3 years after initial treatment. To distinguish between residual stones and recurrence, after lithotomy we performed another imaging study via a balloon catheter as well as peroral cholangioscopy in all patients to confirm and remove any remaining stones and to reduce the risk of residual stones [21]. Virtually all cases of recurrence in this study were considered true recurrence rather than residual stones.

Various factors, such as the parapapillary diverticulum and the diameter of the common bile duct, have been implicated in stone recurrence [8,19,22], and two or more episodes of recurrence frequently occur in the same patient. Prior identification of patients at greatest risk for recurrence allows such patients to be closely followed up at regular intervals. We therefore focused our attention on the role of biliary bacteria as a potential indicator of the risk of recurrence.

Even when a sterilized catheter is used, it is difficult to maintain completely sterile conditions during the transpapillary procurement of bile samples because of contact between the forceps tip and duodenal fluid. Many reports have therefore proposed techniques for biliary surgery or percutaneous transhepatic cholangio-drainage (PTCD) that can be performed aseptically. However, as for techniques for measuring bacteria, although some prior studies have counted the number of isolates or semi-quantitatively estimated bacterial quantity, few have quantified the amount of bacteria [23–27]. We used a scope, which had been mechanically washed, and a cannula to obtain samples of bile as aseptically as possible.

Bacteriological examinations revealed no bacteria in 10 of the 13 patients in the control group. Organisms were detected in the other three patients, but the bacterial counts were all less than 2.2 log CFU/ml. Technically, therefore, bile samples were apparently able to be taken under relatively aseptic conditions. This suggests that when the bacterial count was similar or less than that in the control group, the detected bacteria were a result of contamination occurring at the time of catheter placement.

In the 17 patients in whom several bile samples were taken at different times, more bacterial isolates were detected after lithotomy than before EST, confirming that opening the peripheral part of the common bile duct was associated with the presence of bacteria in the bile soon after the procedure. The bacterial count on follow-up examinations performed 3 months or more after lithotomy was slightly, but not significantly, lower than that before EST. Therefore, although after lithotomy the EST-induced improvement in jaundice and the systemic administration of antibiotics may decrease the bacterial count, opening of the common bile duct resulted in the early adherence of bacteria to the inside wall of the bile duct, and substantial quantities of bacteria persisted even at the time of follow-up. Previous studies have also reported the adherence of bacteria to the inside of the bile duct soon after incision of the common bile duct by EST [24,25,28,29] as well as the early detection of bacteria in bile soon after drain insertion in patients undergoing endoscopic retrograde biliary drainage [18].

When the bile bacteria at follow-up were compared between recurrent and nonrecurrent cases, gram-negative bacilli such as Klebsiella and E. coli and Enterobacilli such as Enterococcus were frequently isolated in both groups, and Streptococcus, Enterobacter, and Pseudomonas were relatively frequently detected in patients without recurrence. Multiple isolates were found in 100% of the patients with recurrence and in 80.6% of patients who were followed up. Multiple isolates often included gram-negative bacilli. In patients with recurrence, multiple isolates included Klebsiella in 80% of cases and E. coli in 40%. There was no difference between the groups in anaerobes. The mean bacterial count was significantly higher in the patients with recurrence ($7.4 \pm 0.7$ log CFU/ml) than in those without recurrence ($4.9 \pm 1.8$ log CFU/ml).

When bile samples are taken perorally, a bacterial count of $10^4$ CFU/ml has been reported to indicate the presence of a pathogen responsible for biliary tract infection [30]. In our study, however, follow-up examinations performed 3 months or more after lithotomy, when inflammation and clinical signs and symptoms had already resolved, showed a mean bacterial count of 4.8 log CFU/ml, indicating the presence of substantial amounts of bacteria.

On cholescintigraphic and bacteriologic examinations of the bile, the mean number of isolates was $2.6 \pm 1.1$, and the mean bacterial count was $3.2 \pm 1.1$ log CFU/ml even in patients with good bile passage. These high values indicate that even in the presence of bacteria in bile there is minimal risk of establishing a site for inflammation, provided that biliary flow and passage are good. EST performed via medium or large incisions has the risk of retrograde infection. Most institutions therefore minimize incision size. However, at our institution the incidence of recurrent choledolithiasis, a late complication of EST, was about 8.0%, which is not higher than the rate at other institutions. This finding indicates that incision length has minimal effect on the incidence recurrence. Shigeno *et al.* [18] reported that the bacterial concentration of bile is in the range of $10^4$–$10^6$ CFU/ml when the bile flow is 300 ml/day or more. Conversely, a bile flow of 150 ml/day or less is associated with a rise in the bacterial count to more than $10^8$ CFU/ml. Therefore, bile flow appears to be lower in patients with recurrence than in those without recurrence.

Whether or not biliary bacteria express $\beta$-glucuronidase activity is related to the bacterial quantity as well as the intensity of $\beta$-glucuronidase activity; the presence of $\beta$-glucuronidase inhibitors has also been reported [9–13]. There is also positive correlation between total bile acid concentration and $\beta$-glucuronidase inhibition rate [9–13].

Cholescintigraphy revealed significantly more biliary isolates and higher bacterial counts in patients with poor bile passage than those with good passage. In addition, both patients with recurrence had delayed passage. These findings indicate that $\beta$-glucuronidase activity in patients with delayed passage is higher than that in patients with good passage, creating an environment conducive to the formation of calcium bilirubinate stones in the former group. Especially when the bacterial count is higher than 7.0 log CFU/ml, the risk of a decrease in bile flow and an increased stone recurrence would possibly be found. Bile acid preparations, used clinically as choleretic agents, increase the total bile acid concentration as well as the bile-acid-dependent bile concentration, favoring a decreased concentration of bacteria.

Increases in the number of isolates and the bacterial count, seen in patients with recurrence, are important factors in stone recurrence. These findings suggest a decrease in bile flow and indicate an increased risk of recurrence. Patients presenting with these findings should therefore be carefully followed by ultrasonography or ERCP at regular intervals.

### 5. CONCLUSIONS

Biliary bacterial examinations and cholescintigraphy were performed in patients with choledocholithiasis. The following results were obtained:

(1) No bacteria were detected in 10 of the 13 patients in the control group. In three patients the bacterial count was 2.2 log CFU/ml or less.
(2) Adherence of bacteria to the bile duct was seen soon after opening the peripheral part of the common bile duct. High concentrations of bacteria were found even on follow-up examinations.
(3) The number of isolates and bacterial quantity in patients with recurrence were slightly higher than those in patients with no recurrence.
(4) Cholescintigraphy revealed a trend toward a higher number of isolates and a higher bacterial count in bile in patients with delayed bile passage than in those with good passage.

The results suggested that an increased number of biliary isolates and an increased bacterial count indicate decreased bile flow in patients with choledocholithiasis who are being followed up after EST. In the case of more than 7.0 log CFU/ml in bacterial count could cause the high risk of stone recurrence. These variables may potentially serve as indicators of the risk of stone recurrence.

### *Acknowledgment*

On the completion of this manuscript, I would like to thank Prof. Yoshihiro Sakai of the Department of Gastroenterologic Endoscopy, Toho University School of Medicine for his guidance throughout this study, Prof. Keizo Yamaguchi of the Department of Microbiology for performing the bacteriological examinations, and to Dr. Yoshinori Igarashi, Lecturer, Third Department of Internal Medicine, Toho University School of Medicine, for his helpful advice. I would also like to thank my colleagues of the Third Department of Internal Medicine as well as Mitsubishi Chemicals Co., Ltd. for their cooperation in bile bacteria culture.

The major findings of this study were reported at DDW-Japan 1995 (panel discussion at the 31st General Meeting of the Japan Biliary Association) and at the 10th International Workshop on Therapeutic Endoscopy, DDW-Japan 1996 (32nd General Meeting of the Japan Biliary Association).

### *References*

[1] Classen, M. and Demling, L. Endoskopiche Sphinkterotomie der Papilla Vateri und Steinextraktion aus dem Duktus Choledochus. *Dtsch. Med. Wschr.* 1974; **99**: 496–497 (in German).
[2] Sohma, S., Tatekawa, I., Okamoto, Y. *et al.* Endoscopic papillotomy: A new approach for extraction of residual stones. *Gastroenterol. Endosc.* 1974; **16**: 446–452 (in Japanese).
[3] Kawai, K., Akasaka, Y., Hashimoto, Y. *et al.* Preliminary report on endoscopical papillotomy. *J. Kyoto Pref. Univ. Med.* 1973; **82**: 353–355.
[4] Bergman, J.G.H.M., van der Mey, S., Rauws, E.A.J. *et al.* Long-term follow-up after endoscopic sphincterotomy for bile duct stones; report on 100 patients with a median follow-up pf 15 years. *Am. J. Soc. Gastrointestinal. Endosco.* 1996; **44**: 643–649.
[5] Hawes, R.H., Cotton, P.B. and Vallon, A.G. Follow-up 6 to 11 years after duodenoscopic sphincterotomy for stones in patients with prior cholecystectomy. *Gastroenterology* 1990; **98**: 1008–1012.
[6] Tanaka, M., Ikeda, S., Yoshimoto, H. *et al.* The long-term fate of the gallbladder after endoscopic sphintectomy. *Am. J. Surg.* 1987; **154**: 505.
[7] Ogawa, Y., Tanaka, M., Ikeda, S. *et al.* Treatment of common bile duct stones by endoscopic sphincterotomy (ES) – Apprasal of ES for stones in young patients and migrating from the gallbladder. *Bil. Tract and Panc.* 1995; **16**: 301–305 (in Japanese).
[8] Hajime, H. and Yoshihiro, S. Clinical study on causative factors and recurrence of choledocholithiasis. *Diagnostic and Therapeutic Endosco.* 1996; **3**: 81–91.
[9] Maki, T. Pathogenesis of calcium bilirubinate gall stone: Role of E. coli, β-glucuronidase and coagulation by inorganicions, polyelectrolytes and agitalion. *Ann. Surg.* 1996; **164**: 90–100.
[10] Turley, S.D. and Dietschy, J.M. Reevaluation of the 3-hydroxysteroid dehydrogenase assay for total bile acids in bile. *J. Lipid Res.* 1978; **19**: 945–955.
[11] Gurantz, D., Laker, M.F. and Hofmann, A.F. Enzymatic measurement of choline containing phospholipids in bile. *J. Lipid Res.* 1981; **22**: 379–391.
[12] Ho, Y.C. and Ho, K.J. Human β-glucuronidase. Measurement of its activity in gallbladder bile devoid of intrinsic interference. *Dig. Dis. Sci.* 1988; **33**: 435–442.

[13] Ho, Y.C. and Ho, K.J. Differential quantitation of urinary $\beta$-glucuronidase of human and bacterial origins. *J. Urol.* 1985; **134**: 1227–1230.

[14] Tamotsu, A. Clinical assessment of endoscopic naso-biliary drainage (ENBD) for the prevention of cholangitis and bile congestion associated with endoscopic lithotoripsy of common bile duct stones. *Gastroenterol. Endosc.* 1994; **36**: 1175–1185 (in Japanese).

[15] Yamagata, K., Maki, T., Osuga, T. *et al.* New classification for gallstones in Japan. *J. J. Gastroenterol.* 1986; **83**: 309–316 (in Japanese).

[16] Kuniyasu, Y. Cholescintigraphy using $^{99m}$TC-HIDA. In: Hiramatu, K. (Ed.) *New Atlas of Anatomy for Diagnostic Imaging*. Tokyo Medical View Ltd. 1990 pp. 122–125 (in Japanese).

[17] Tanaka, M., Ogawa, Y., Naritomi, G. *et al.* Clinical significance of endoscopic sphincterotomy compared with surgical common bile duct exploration and surgical sphincterotomy. *J. Jpn. Surg. Soc.* 1992; **93**: 1119–1122 (in Japanese).

[18] Shigeno, T. Bacterial characteristics of bile in patients treated with endoscopic retrograde biliary drainage (ERBD). *Gastroenterol. Endosco.* 1990; **32**: 334–344 (in Japanese).

[19] Takahashi, W., Suzuki, N., Uematsu, I. *et al.* Recurrent stone in the aspect of perivaterian diverticurum. *Jpn. J. Soc. Gastroenterol. Surg.* 1983; **16**: 762–767 (in Japanese).

[20] Takekawa, K. and Hasebe, O. Late complication of endoscopic sphincterotomy. *J. Biliary Assosiation* 1995; **9**: 136 (in Japanese).

[21] Igarashi, Y., Hasegawa, T., Anzai, T. *et al.* Clinical evaluation of endoscopic lithotomy for stonesin the common bile duct. *Endoscopia Digestiva* 1993; **5**: 909–914 (in Japanese).

[22] Adachi, W., Yamagishi, K., Kajikawa, S. *et al.* Clinical significances of juxtapapillary duodenal diverticula. *Jpn. J. Soc. Gastroenterol. Surg.* 1986; **19**: 2030–2034 (in Japanese).

[23] Gregg, J.A., De Girolami, P. and Carr Locke, D.L. Effects of spincteroplasty and endoscopic sphincterotomy on the bacteriologic characteristics of the common bile duct. *Am. J. Surg.* 1985; **149**: 668–671.

[24] Sand, J., Airo, I., Hiltunen, K.M., Mattila, J. *et al.* Changes in biliary bacteria after endoscopic cholangiography and sphincterotomy. *Am. Surg.* 1992; **58**: 324–328.

[25] Skar, V., Skar, A.G., Midtvedt, T. *et al.* Bacterial growth in the duodenum and in the bile of patients with gallstone disease treated with endoscopic papillotomy. *Endoscopy.* 1986; **18**: 10–13.

[26] Kido, K., Nakajima, Y., Kanehiro, H. *et al.* A study of intraoperative and postoperative culture of bile juice in the cases undergoing biliary tract operation. *J. Jpn. Soc. Clin. Surg.* 1993; **54**: 2754–2759 (in Japanese).

[27] Kosugi, H., Higuchi, T., Yoshinaga, T. *et al.* A study of isolated organisms in bile directly collected by endoscopic cannuration of the papilla of Vater. *J. J. Biliary Assosiation* 1987; **1**: 372–376 (in Japanese).

[28] Ishiguro, J., Igarashi, Y. and Sakai, Y. Bacteriological study of bile after endoscopic shincterotomy (EST). *J. J. Biliary Assosiation* 1995; **9**: 137 (in Japanese).

[29] Bergman, J.G.H.M., Anne-Marie van Berkel, Albert K. Groen *et al.* Biliary manometry, bacterial characteristics, bile composition, and histologic changes fifteen to seventeen years after endoscopic sphinterotomy. *Am. J. Soc. Gastrointestinal. Endosco.* 1997; **45**: 400–405.

[30] Shinagawa, N., Suzuki, K., Suzuki, Y. *et al.* Clinical significance of bacterial flora in biliary tract disease. *Jpn. J. Soc. Gastroenterol. Surg.* 1983; **16**: 543–546 (in Japanese).

